# Lack of SLC26A9-mediated chloride secretion causes mucus plugging and severe respiratory distress in neonatal mice

**DOI:** 10.1172/jci.insight.196355

**Published:** 2025-10-16

**Authors:** Pamela Millar-Büchner, Johanna J. Salomon, Julia Duerr, Stephan Spahn, Pinelopi Anagnostopoulou, Willi L. Wagner, Mark O. Wielpütz, Hermann-Josef Groene, Anita Balázs, Marcus A. Mall

**Affiliations:** 1 Department of Pediatric Respiratory Medicine, Immunology and Critical Care Medicine, Charité - Universitätsmedizin Berlin, Berlin, Germany.; 2 German Center for Lung Research (DZL), associated partner site Berlin, Berlin, Germany.; 3 Department of Translational Pulmonology, University Hospital Heidelberg, Heidelberg, Germany.; 4 Translational Lung Research Center Heidelberg (TLRC), German Center for Lung Research (DZL), Heidelberg, Germany.; 5 German Center for Child and Adolescent Health (DZKJ), partner site Berlin, Berlin, Germany.; 6 Medical School, University of Cyprus, Nicosia, Cyprus.; 7 Department of Diagnostic and Interventional Radiology, University Hospital Heidelberg, Heidelberg, Germany.; 8 Institute of Diagnostic Radiology and Neuroradiology, University Medicine Greifswald, Greifswald, Germany.; 9 Department of Cellular and Molecular Pathology, German Cancer Research Center (DKFZ), Heidelberg, Germany.; 10 Institute of Pharmacology, University of Marburg, Marburg, Germany.; 11 Cluster of Excellence ImmunoPreCept, Charité - Universitätsmedizin Berlin, Berlin, Germany.

**Keywords:** Development, Inflammation, Pulmonology, Epithelial transport of ions and water, Ion channels

## Abstract

Solute carrier family 26, member 9 (SLC26A9) is an epithelial chloride channel that was identified as a genetic modifier of disease severity of cystic fibrosis (CF) and other chronic muco-obstructive lung diseases. However, data on the in vivo role of SLC26A9 function in lung health and disease remain limited. Here, we investigated the effect of genetic deletion of *Slc26a9* (*Slc26a9^–/–^*) on the pulmonary phenotype of neonatal mice. We found that lack of *Slc26a9* causes severe neonatal respiratory distress with high mortality. Histology, immunohistochemistry, and micro-computed tomography imaging studies identified airway obstruction with MUC5B-positive mucus plugs in neonatal *Slc26a9^–/–^* mice. Bioelectric measurements demonstrated a reduced transepithelial potential difference indicative of reduced chloride secretion across tracheal explants of neonatal *Slc26a9^–/–^* compared with WT mice. In addition, neonatal *Slc26a9^–/–^* mice displayed hypoxic degeneration of airway epithelial cells associated with sterile neutrophilic airway inflammation. Collectively, our data show that SLC26A9-mediated chloride secretion is critical for proper mucociliary clearance, respiratory function, and survival after birth, and identify a role for SLC26A9 in neonatal adaptation during the transition from fetal to neonatal life.

## Abstract 2

Lack of SLC26A9-mediated chloride secretion causes airway mucus plugging and severe respiratory distress in neonatal mice

## Graphical abstract

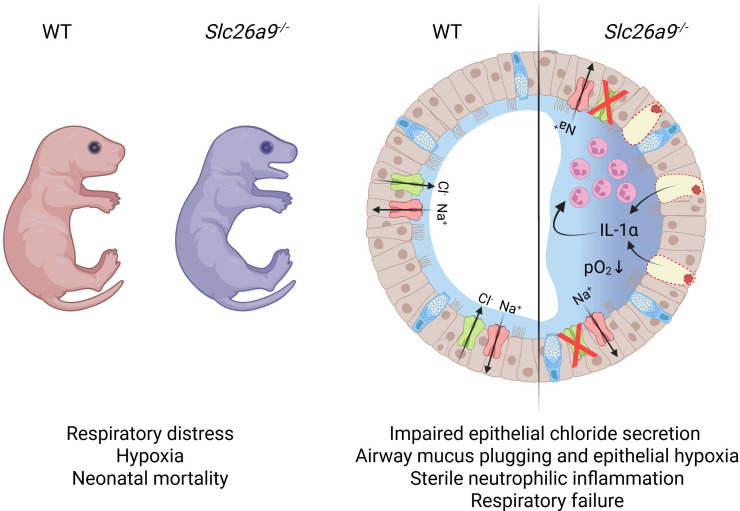

## Introduction

Solute carrier family 26, member 9 (SLC26A9) is a constitutively active chloride transporter with important roles in coordinated ion and fluid transport in the lung (1, 2). Single-cell RNA sequencing studies identified *SLC26A9* expression in the lung epithelium, predominantly in secretory and alveolar type 2 cells, where it overlaps considerably with the expression of the cystic fibrosis transmembrane conductance regulator (*CFTR*) chloride channel (3). Functional studies demonstrated that SLC26A9 mediates constitutive chloride secretion in the airways (4–6), whereas it may also augment CFTR-mediated chloride secretion by promoting its trafficking and/or by stimulatory interactions (7–9). The importance of SLC26A9 in human lung physiology was highlighted by a series of studies demonstrating a genetic association between *SLC26A9* polymorphisms and a spectrum of muco-obstructive lung diseases, including cystic fibrosis (CF), non-CF bronchiectasis, and asthma (3, 10–12). In patients with CF, *SLC26A9* variants have also been reported to modify response to CFTR modulator therapies (3). Furthermore, a strong association was detected between *SLC26A9* single-nucleotide polymorphisms and lung function in the general population, supporting the notion that SLC26A9 may contribute to the etiology of airflow obstruction (3, 13).

In a previous study elucidating the in vivo function of SLC26A9 in adult mice, we showed that SLC26A9 does not contribute to airway chloride secretion and is not essential for lung health under physiological conditions (10). However, we found that SLC26A9-mediated chloride secretion is upregulated in parallel with increased mucin expression in type 2 airway inflammation, and that this coordinated upregulation of chloride/fluid and mucin secretion is essential to prevent airway mucus plugging in a mouse model of experimental asthma (10). These results support the concept that SLC26A9-mediated chloride secretion plays an important role in maintaining adequate airway surface hydration and thus in preventing mucus hyperconcentration, which leads to impaired mucociliary clearance and mucus plugging in the context of mucus hypersecretion in inflamed airways (10, 14, 15). Despite the lack of a lung disease phenotype in unchallenged adult *Slc26a9*-deficient (*Slc26a9^–/–^*) mice (10), another study observed that *Slc26a9* deficiency is associated with high neonatal mortality in the absence of inflammatory stimuli; however, the underlying cause of early death remains unknown (16).

The aim of this study was therefore to determine the role of SLC26A9 in homeostatic postnatal adaptation, during the transition from a predominantly secretory fetal lung epithelium to an epithelium that facilitates net absorption of salt and water to facilitate clearance of fetal lung liquid and enable air-breathing (17). To achieve this goal, we investigated the effects of genetic deletion of *Slc26a9* on the pulmonary phenotype of newborn mice. Specifically, we monitored survival, studied lung morphology by histology and micro-computed tomography (μCT) imaging, and measured inflammatory cells and cytokine profiles in the lungs of neonatal *Slc26a9^–/–^* mice and wild-type (WT) littermates. Furthermore, we investigated fetal lung liquid clearance, as well as bioelectric properties of tracheal explants of newborn *Slc26a9^–/–^* and WT mice.

## Results

### Genetic deletion of Slc26a9 causes severe respiratory distress with high mortality in neonatal mice.

**Figure 1:**
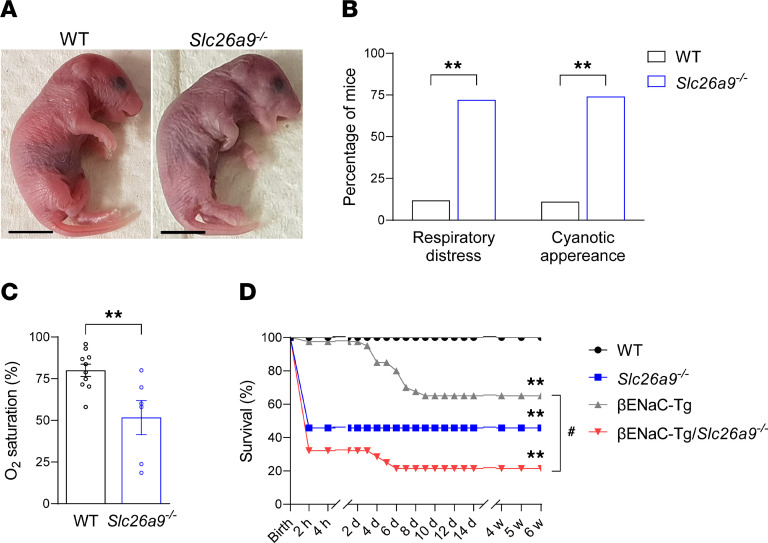
Genetic deletion of *Slc26a9* causes respiratory distress and death in neonatal mice and aggravates mortality of βENaC-Tg mice with muco-obstructive lung disease. (**A**) Appearance of WT and *Slc26a9^–/–^* mice 30 minutes after birth. (**B**) Percentage of newborn WT and *Slc26a9^–/–^* mice developing respiratory distress and/or cyanotic appearance (*n* = 136–282 mice per group). (**C**) Oxygen saturation measurements in newborn WT and *Slc26a9^–/–^* mice (*n* = 6–10 mice per group) 30 minutes to 2 hours after birth. (**D**) Survival curves of WT, *Slc26a9^–/–^*, βENaC-Tg, and βENaC-Tg/*Slc26a9^–/–^* mice (*n* = 21–39 mice per group). **P < 0.01 versus WT mice and ^#^*P* < 0.01 versus βENaC-Tg mice by χ^2^ test (**B**), 2-tailed Student’s *t* test (**C**), and Kaplan-Meier test (**D**).

To evaluate the effect of *Slc26a9* deficiency on postnatal adaptation and survival, we closely monitored newborn pups during the first hours after birth. WT mice established regular breathing and appeared pink within the first 10 minutes of life, whereas nearly 70% of neonatal *Slc26a9^–/–^* mice displayed signs of respiratory distress, such as irregular breathing, gasping and chest wall retractions, cyanotic appearance, and reduced oxygen saturation levels (Figure 1, A–C). Survival analysis revealed approximately 50% mortality in *Slc26a9^–/–^* mice within the first 2 hours after birth (Figure 1D). Notably, no further survival disadvantage was seen up to the age of 6 weeks in *Slc26a9^–/–^* mice. In addition, we evaluated the effect of *Slc26a9* deficiency on the survival of βENaC-Tg mice, an established model of early-onset muco-obstructive lung disease (18, 19). In line with previous studies, βENaC-Tg mice displayed approximately 30% mortality from postnatal day 3 (P3) onward (19). Genetic deletion of *Slc26a9* strongly enhanced the onset and severity of mortality in βENaC-Tg mice (Figure 1D). Due to the high level of mortality, no further analysis was performed in *Slc26a9^–/–^* βENaC-Tg mice. At birth, all genotypes were represented according to the expected Mendelian ratio (data not shown).

### Neonatal Slc26a9^–/–^ mice display severe airway mucus obstruction.

**Figure 2:**
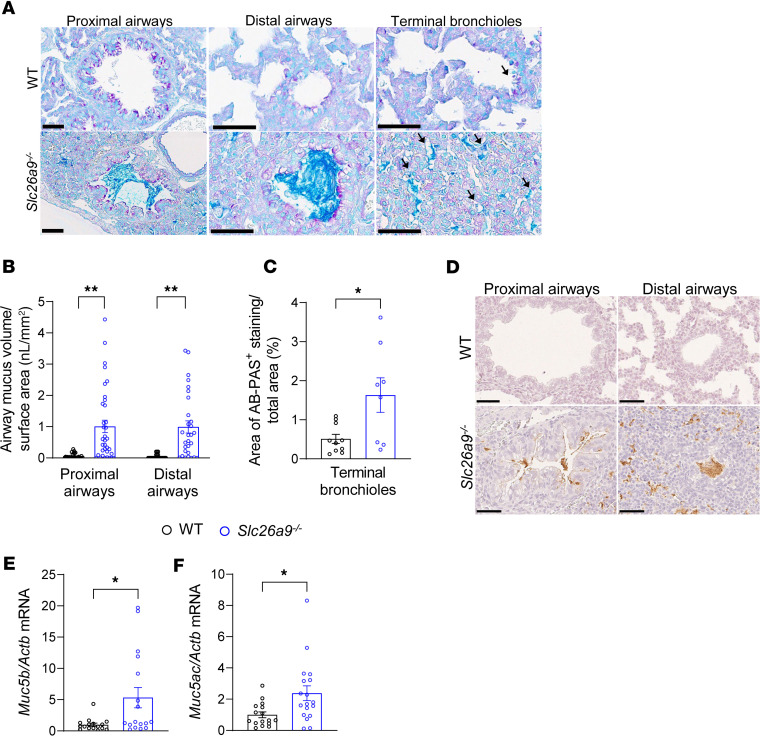
Genetic deletion of *Slc26a9* causes airway mucus obstruction in neonatal mice. (**A**) Representative images of AB-PAS–stained lung sections showing proximal and distal conducting airways and terminal bronchioles (arrows) of neonatal WT and *Slc26a9^–/–^* mice. Scale bars: 50 μm. (**B** and **C**) Summary of airway mucus volume density in the main proximal and distal airways (**B**) and percentage of total area of the terminal bronchioles obstructed with mucus (**C**) in neonatal WT and *Slc26a9^–/–^* mice (*n* = 27–35 mice per group). (**D**) Representative images of MUC5B immunohistochemistry in proximal and distal airways of WT (*n* = 3) and *Slc26a9^–/–^* (*n* = 5) mice. Scale bars: 50 μm. (**E** and **F**) Transcript analysis of secreted mucins *Muc5b* and *Muc5ac* in lung homogenates of neonatal WT and *Slc26a9^–/–^* mice. *P < 0.05, **P < 0.01 versus WT mice by Mann-Whitney test (**B**, **C**, and **E**) and 2-tailed Student’s *t* test (**F**).

To investigate the underlying respiratory pathology, we performed histological analysis of the tracheae and lungs of neonatal (P0) *Slc26a9^–/–^* mice and WT littermates. Tissue sections showed no difference in goblet cell numbers in the tracheal epithelium of the 2 genotype groups, but accumulation of Alcian blue–periodic acid–Schiff–positive (AB-PAS–positive) material in the lumen of the trachea, intrapulmonary proximal and distal airways, as well as in the terminal bronchioles of neonatal *Slc26a9^–/–^* mice that was not observed in WT mice (Figure 2, A–C, and Supplemental Figure 1; supplemental material available online with this article; https://doi.org/10.1172/jci.insight.196355DS1). Immunostaining with antibodies against MUC5B showed that this AB-PAS–positive material in neonatal *Slc26a9^–/–^* mice stained positive for MUC5B, whereas little MUC5B-positive staining was observed in lungs of WT mice, thus demonstrating that perinatal lack of SLC26A9 causes airway mucus plugging after birth (Figure 2D). *Muc5b* and *Muc5ac* transcripts were expressed in lung homogenates of both genotype groups, with increased levels in neonatal *Slc26a9^–/–^* compared with WT mice (Figure 2, E and F). To determine whether *Slc26a9* deficiency affected prenatal development, we performed histopathological analysis of vital organs, including the brain, heart, lung, liver, pancreas, and intestine on embryonic day 17 and did not detect any differences between *Slc26a9^–/–^* and WT mice (Supplemental Figure 2).

**Figure 3:**
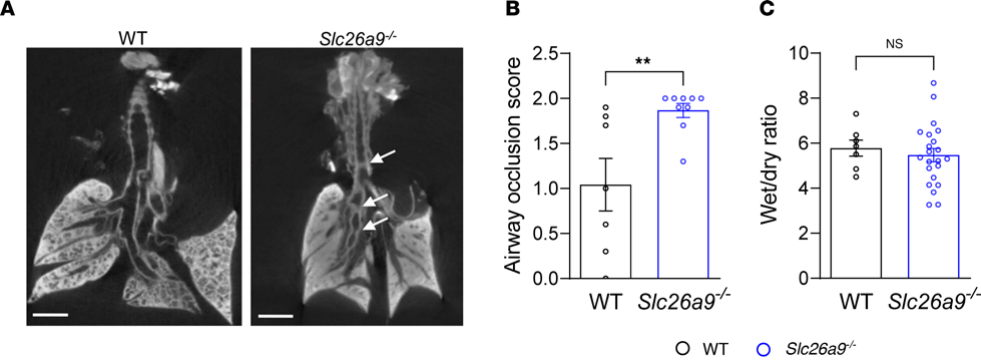
Genetic deletion of *Slc26a9* leads to airway mucus plugging and atelectasis, but does not affect fetal lung liquid clearance. Representative μCT images of the lungs (**A**) and summary of airway occlusion score (**B**) of neonatal WT and *Slc26a9^–/–^* mice. White arrows indicate airway occlusion. Scale bar: 1 mm (*n* = 7–10 mice per group). (**C**) Lung liquid content determined by wet/dry weight ratio in neonatal WT and *Slc26a9^–/–^* mice (*n* = 7–22 mice per group). **P < 0.01 versus WT mice by 2-tailed Student’s *t* test (**B** and **C**).

To assess the extent of airway mucus plugging and other morphological changes in the lungs of neonatal (P0) *Slc26a9^–/–^* mice, we used μCT as a high-resolution 3-dimensional imaging modality (20, 21). The lung parenchyma of neonatal WT mice displayed a homogeneous lung texture and full aeration of the lung, while neonatal *Slc26a9^–/–^* mice showed opacification in all lobules indicating unventilated parenchyma due to atelectasis (Figure 3A and Supplemental Videos 1 and 2). Neonatal *Slc26a9^–/–^* mice also displayed partial or complete opacification of the trachea and bronchi, and a significantly higher airway occlusion score than WT littermates (Figure 3B). To investigate the possibility that opacification of the lung parenchyma may alternatively be caused by retention of alveolar fluid due to impaired clearance of fetal lung liquid, we determined wet-to-dry weight ratios of whole lungs as a parameter that reflects lung liquid content (22). We found no differences in wet-to-dry weight ratios between WT and *Slc26a9^–/–^* mice sacrificed at 30 minutes after birth, thus arguing against impaired lung liquid clearance in neonatal *Slc26a9^–/–^* mice (Figure 3C).

### Changes in transepithelial ion transport in tracheal explants of neonatal Slc26a9^–/–^ mice.

**Figure 4:**
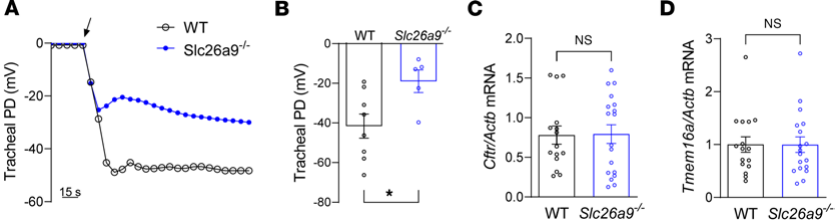
Genetic deletion of *Slc26a9* alters transepithelial ion transport in airways of neonatal mice. (**A** and **B**) Bioelectric properties of the airway epithelium studied by micro-electrode impalement of tracheal explants from neonatal WT and *Slc26a9^–/–^* mice. Representative recordings (**A**) and summary (**B**) of transepithelial potential difference (PD) measurements. The black arrow indicates impalement of the tracheal lumen (*n* = 5–8 mice per group). (**C** and **D**) Transcript levels of *Cftr* (**C**) and *Tmem16a* (**D**) in lung homogenates of WT and *Slc26a9^–/–^* mice (*n* = 15–18 mice per group). *P < 0.05 versus WT mice by 2-tailed Student’s *t* test.

SLC26A9 is a chloride transporter that has been shown to play an important role in coordinated epithelial ion and fluid secretion under conditions of airway inflammation in adult mice (1, 10). To study SLC26A9 function in the neonatal lung, we cultured tracheal explants from *Slc26a9^–/–^* and WT newborn (P0) pups and compared their bioelectric properties using microelectrode impalement studies. These experiments demonstrated that the lumen-negative transepithelial potential difference across the epithelium of tracheal explants of neonatal *Slc26a9^–/–^* mice was significantly reduced compared with WT mice, indicating reduced transepithelial chloride secretion in the airways of neonatal *Slc26a9^–/–^* mice (Figure 3B and Figure 4, A and B). We also investigated the expression of *Cftr* and *Tmem16a*, 2 epithelial chloride channels that mediate cAMP-stimulated and calcium-activated chloride secretion across the airway epithelium, respectively (23–25). We found no difference in mRNA transcript levels of these chloride channels between WT and *Slc26a9^–/–^* lungs, suggesting that genetic deletion of *Slc26a9* did not lead to a compensatory upregulation of either *Cftr* or *Tmem16a* (Figure 4, C and D). In addition, we investigated the expression of *Slc26a9* in WT mouse lungs during development, which showed induction of expression in the first 3 days of life that was sustained into adulthood (Supplemental Figure 3).

### Neonatal Slc26a9^–/–^ mice display epithelial cell degeneration and sterile neutrophil inflammation.

**Figure 5:**
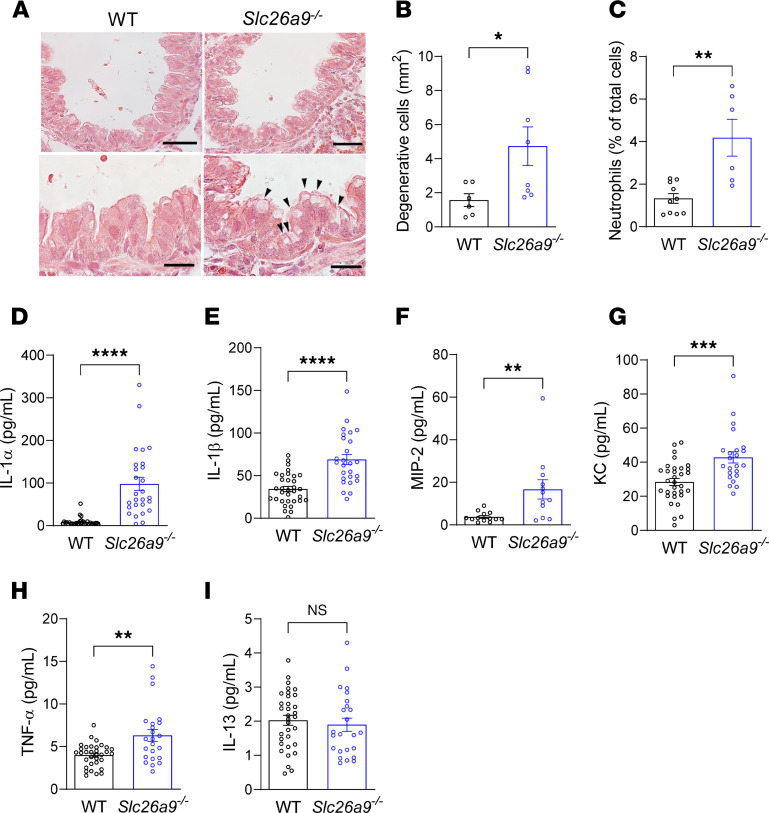
Genetic deletion of *Slc26a9* is associated with airway epithelial cell degeneration and inflammation in neonatal mice. (**A**) Representative airway sections of neonatal WT and *Slc26a9^–/–^* mice stained with H&E. Black arrowheads indicate degenerative cells. Scale bars: 20 μm and 10 μm (insets). (**B**) Quantification of degenerative cells in neonatal WT and *Slc26a9^–/–^* mice (*n* = 6–8 mice per group). (**C**) Quantification of neutrophils in lung homogenates of neonatal WT and *Slc26a9^–/–^* mice (*n* = 6–11 mice per group). (**D**–**I**) Levels of proinflammatory cytokines IL-1α (**D**), IL-1β (**E**), MIP-2 (**F**), KC (**G**), TNF-α (**H**), and IL-13 (**I**) in lung homogenates of neonatal WT and *Slc26a9^–/–^* mice (*n* = 19–31 mice per group). *P < 0.05, **P < 0.01, ***P < 0.001, ****P < 0.0001 versus WT mice by Mann-Whitney test.

Previous studies in βENaC-Tg mice identified a mechanistic link between airway mucus plugging, hypoxic epithelial necrosis, and neutrophilic inflammation, which are key features of this model of muco-obstructive lung disease (26, 27). To determine whether airway mucus plugging in neonatal *Slc26a9^–/–^* mice also triggered airway epithelial necrosis and inflammation, we evaluated lung sections for necrotic airway epithelial cells and determined inflammatory cell counts and cytokine profiles in the lungs of neonatal (P0) *Slc26a9^–/–^* compared with WT mice. Histologic analysis of hematoxylin and eosin–stained (H&E-stained) airway sections revealed an increased number of hydropic, degenerative cells, i.e., characteristic features of hypoxic necrosis (28), in the airway epithelium of newborn *Slc26a9^–/–^* mice (Figure 5, A and B). Flow cytometric analysis showed an increase in neutrophils in the lungs of neonatal *Slc26a9^–/–^* versus WT mice (Figure 5C), whereas other inflammatory cell types did not differ between genotypes (data not shown). Furthermore, we found a marked (~11-fold) increase in the concentration of the proinflammatory cytokine IL-1α that is released from necrotic cells (29, 30), as well as a significant increase in the levels of IL-1β, TNF-α, and the neutrophil chemoattractants KC and MIP-2 in the lungs of neonatal *Slc26a9^–/–^* mice versus WT mice. The Th2 cytokine IL-13 did not differ between genotypes (Figure 5, C–I). To determine a potential role of bacterial infection as a trigger for pulmonary inflammation observed in neonatal *Slc26a9^–/–^* mice, we submitted homogenates for microbiological analysis. No bacterial growth was observed in cultures of lung homogenates of neonatal WT or *Slc26a9^–/–^* mice (data not shown).

## Discussion

The epithelial chloride channel SLC26A9 has been implicated as a modifier of chronic muco-obstructive lung diseases such as CF, bronchiectasis, and asthma (3, 10, 11); however, its role in the transition from fetal to neonatal life has not been studied. This study demonstrates for the first time to our knowledge that genetic deletion of *Slc26a9* producing reduced transepithelial chloride secretion leads to severe neonatal respiratory distress with high mortality. We found that this neonatal respiratory distress syndrome is caused by airway mucus obstruction associated with airway inflammation. Collectively, our data identify an important role of SLC26A9 for proper mucociliary clearance that is essential for neonatal respiratory function and adaptation during the transition from fetal to neonatal life.

The original report of the *Slc26a9^–/–^* mouse evaluated the gastric phenotype and did not report early mortality of this model, but did also not provide information on how the observed genotype frequencies compared to the expected Mendelian ratios (31). A follow-up study on the role of SLC26A9 in the intestine using mice on the same genetic background (S129/svj) reported high neonatal mortality of *Slc26a9^–/–^* mice in the first days of life; however, the underlying cause of early death remained unknown (16). We backcrossed *Slc26a9^–/–^* mice to the C57BL/6J background and also observed high neonatal mortality. Based on the clinical phenotype of severe respiratory distress after birth and the lack of prenatal histomorphological changes of vital organs, including the brain, heart, lung, liver, and intestine in *Slc26a9^–/–^* mice, we speculated that respiratory failure may be caused by impaired lung liquid clearance. The fetal lung is filled with liquid, which is rapidly reabsorbed postnatally. This process is primarily driven by the epithelial sodium channel ENaC that is expressed in lung epithelial cells from late fetal gestation where it reaches near-adult levels on the first day of life (32). Expression of ENaC leads to active transcellular absorption of sodium that generates an electrochemical driving force for paracellular or transcellular absorption of chloride and water (33), and genetic deletion of the α-subunit of ENaC that is essential to form functional sodium channels was shown to cause neonatal respiratory distress and death due to defective lung liquid clearance (22). While SLC26A9 chloride channels are coexpressed with ENaC in lung epithelial cells, their role in the lung parenchyma has not been studied. We hypothesized that SLC26A9 may act as an absorptive chloride channel in the alveoli and thus contribute to the clearance of fluid from the airspaces. However, in our measurements of wet-to-dry weight ratios of lungs, we found no differences between neonatal *Slc26a9^–/–^* and WT mice. These data argue against a role of impaired liquid clearance as the underlying cause of neonatal respiratory distress in *Slc26a9^–/–^* mice.

However, our histological and μCT imaging studies revealed a striking obstruction of the conducting airways of neonatal *Slc26a9^–/–^* mice by mucus plugs. The presence of mucus obstruction in AB-PAS–stained sections and mucus plugging of proximal and distal conducting airways associated with opacities of the parenchyma distal to the plugs, as shown by μCT imaging, suggests that the parenchyma collapses because it is not ventilated and inflated with air during the transition to air breathing and therefore becomes atelectatic when fetal lung liquid is absorbed. Furthermore, we found that the lumen-negative transepithelial potential difference across tracheal explants was significantly reduced in *Slc26a9^–/–^* compared with WT mice. This change in lumen-negative potential difference is consistent with reduced transepithelial chloride secretion. Collectively, these data support the notion that SLC26A9 is implicated in chloride/fluid secretion rather than absorption, that SLC26A9-mediated chloride secretion is essential for effective hydration and clearance of mucins that are abundantly expressed and constitutively secreted in the neonatal lung (34, 35), and that failure of this process leads to severe airway mucus plugging and neonatal respiratory distress. This concept is supported by findings in patients with CF, where impaired mucus hydration due to deficient CFTR-mediated chloride secretion was identified as a key disease mechanism of mucociliary dysfunction and mucus plugging (15, 36), as well as our data from the crossing of *Slc26a9^–/–^* with βENaC-Tg mice showing that the lethality of neonatal respiratory distress caused by *Slc26a9* deficiency is further exacerbated when airway surface hydration is decreased due to overexpression of ENaC (18).

Our data also provide insights into the link between airway mucus plugging and inflammation in the neonatal lung. Previous studies in the βENaC-Tg mouse as a model of muco-obstructive lung disease showed that mucus plugging can cause local epithelial hypoxia leading to hypoxic cell stress and necrosis, which has been identified as an important trigger for sterile inflammation (26, 28–30). In these studies, it was found that airway epithelial cells undergoing hypoxic degeneration release IL-1α as a preformed danger-associated molecular pattern (DAMP), which activates the IL-1 receptor signaling pathway leading to neutrophilic inflammation that causes structural lung damage in the absence of bacterial infection (26). In the present study, we detected increased numbers of degenerative airway epithelial cells scattered throughout the conducting airways of neonatal *Slc26a9^–/–^* mice, with identical morphology to those found in βENaC-Tg mice. Given the severe mucus plugging in *Slc26a9^–/–^* mice, we assume that epithelial necrosis in *Slc26a9^–/–^* mice is also caused by luminal hypoxia, which is supported by a similar inflammatory response, including elevated IL-1α, IL-1β, and airway neutrophilia that is associated with the appearance of these degenerative cells in βENaC-Tg mice. Furthermore, in vitro studies showed that activation of IL-1 receptor signaling is also a potent trigger of MUC5B production, and that epithelial hypoxia leads to increased production of MUC5B and increased transepithelial sodium and fluid absorption via upregulation of ENaC, leading to hyperconcentrated mucus in human bronchial epithelial cultures, which is predicted to cause mucus stasis and plugging (37, 38). Our findings of hypoxic epithelial degeneration, neutrophilia, elevated IL-1α, and other markers of neutrophilic inflammation in *Slc26a9^–/–^* pups support the notion that this pathogenetic cascade of mucus plugging, epithelial hypoxia, and IL-1 receptor–mediated inflammation can be operative in the neonatal lung immediately after birth and in the absence of detectable infection. This inflammatory response, specifically hypoxia- and IL-1–induced signaling, may play a key role in triggering increased production of MUC5B (34, 35), which may be released from epithelial cells in response to potent triggers of mucus secretion, such as neutrophilic elastase or ATP (39, 40), thereby aggravating mucus hyperconcentration and plugging that causes severe neonatal respiratory distress.

In humans, respiratory distress syndrome remains a leading cause of neonatal morbidity and mortality that commonly results from deficiency of pulmonary surfactant in preterm infants (41). However, twin cohort studies reported that up to 50% of variance in liability to develop respiratory distress syndrome in preterm as well as term infants may be attributed to genetic factors (42, 43). These studies thus indicate that there is a significant genetic susceptibility to develop respiratory distress syndrome; however, the underlying genetic factors remain to be elucidated. In this context, our data in *Slc26a9^–/–^* mice support evaluation of SLC26A9 as a genetic modifier or cause of respiratory distress syndrome in infants, which may be facilitated by the current widespread implementation of next-generation whole-exome and whole-genome sequencing. Of note, the clinical relevance of mucociliary dysfunction in the pathogenesis of neonatal respiratory distress observed in our murine study is supported by the phenotype of patients with primary ciliary dyskinesia (PCD) in whom mucociliary clearance is impaired due to ciliary dysfunction (44). The earliest disease manifestation occurs in the neonatal period, where up to 80% of full-term neonates with PCD present with respiratory distress, including tachypnea and dyspnea commonly requiring oxygen therapy (45).

The spatiotemporal regulation of SLC26A9 expression is largely unexplored. Chromatin state predictions in the human lung (12) and our mouse data suggest that *SLC26A9* expression increases after birth and remains expressed through adulthood. Regarding regulation of SLC26A9-mediated chloride secretion, our functional studies in tracheal explants are consistent with previous work showing that SLC26A9 is constitutively active in the airway epithelium, and in contrast to CFTR or TMEM16A, does not rely on activation by cAMP- or Ca^2+^-elevating agonists (1, 5, 23, 46). In addition to its role as a constitutively active chloride channel, our previous work in adult mice showed that SLC26A9-mediated chloride secretion can be increased by type 2 cytokines to protect mice from airway mucus plugging in allergic airway inflammation (10). Taken together, these data support an important role of SLC26A9-mediated chloride secretion in facilitating effective mucociliary clearance in health and under muco-inflammatory conditions from the neonatal period and later in life.

In summary, our data demonstrate that SLC26A9-mediated chloride secretion is essential for proper mucus clearance and respiratory function during the transition from fetal to neonatal life, and that a failure of this process causes severe neonatal respiratory distress in mice. Furthermore, our data suggest that mucus plugging causes epithelial hypoxia as a potent trigger of inflammation in the neonatal lung in the absence of infection. These data support further studies on the role of SLC26A9 as a genetic modifier and potential therapeutic target in neonatal respiratory distress syndrome and other neonatal lung conditions associated with mucus plugging and inflammation.

## Methods

### Sex as a biological variable.

Our study examined male and female animals, and similar findings are reported for both sexes.

### Study approval.

All animal studies were approved by the animal welfare authority responsible for the University of Heidelberg (Regierungspräsidium Karlsruhe, Germany).

### Experimental animals.

*Slc26a9^–/–^* mice were backcrossed for 10 generations onto the C57BL/6J background and experimental animals were generated from *Slc26a9^+/–^* breeding pairs. Genotyping was performed as previously described (47). Mice were housed in a specific pathogen–free animal facility at Heidelberg University under standardized light cycles and had free access to food and water. To monitor the birth of the pups, full-term pregnant dams (after a gestation period of 19–20 days) were checked every half hour. End-point experiments were performed between 30 minutes and 2 hours after birth on P0, unless specified otherwise. Pups were continuously and regularly monitored from birth up to the age of 6 weeks. The phenotype of each mouse at birth was annotated and classified as regular/irregular breathing and non-cyanotic/cyanotic appearance.

### Oxygen saturation measurements.

Oxygen saturation was determined at room air using a noninvasive pulse oximeter for laboratory animals (MouseOx Plus, STARR Life Science Corporation). Arterial blood oxygen saturation was recorded with a thigh clip sensor.

### Histology and airway morphometry.

Lungs were removed through a median sternotomy, immersion fixed in 4% buffered formalin and embedded in paraffin. Left lungs were sectioned at the level of the proximal intrapulmonary main axial airway near the hilus. Sections were cut at 5 μm and stained with H&E or AB-PAS as previously described (28). Images were acquired with an Olympus IX-71 microscope interfaced with an SIS Colorview I Camera Set (Olympus). For quantitative stereological assessment of mucus obstruction of proximal and distal airways, Cell^F analysis software (Olympus) was used as previously described (28, 48). Briefly, the length of the airway boundary, defined as the epithelial basement membrane, was measured using the interactive image measurement tool, and the AB-PAS–positive surface area within the boundary was measured by phase analysis according to the automatic threshold settings of the software. The volume density of airway mucus, representing the volume of airway mucus content per surface of the basement membrane (nL/mm^2^), was determined from the surface area of AB-PAS–positive mucus and the basement membrane length. Quantification of the mucus obstruction in the terminal bronchiole was performed by using the open source image processing program ImageJ 1.52a (NIH). The threshold was adjusted to determine the percentage of AB-PAS–positive area staining and normalized to the total area of the specimen in the field of view. Degenerative airway epithelial cells were identified using H&E slices by morphologic criteria (i.e., cell swelling with cytoplasmic vacuolization), and numeric cell densities were quantitated by counting epithelial cells per millimeter of the basement membrane (28).

### Immunohistochemistry.

Immunohistochemistry was performed using polyclonal antibody rabbit anti-mouse MUC5B at a dilution of 1:2000 in PBS (H-300, Santa Cruz Biotechnology). Formalin-fixed, paraffin-embedded 1.5 μm left lobe sections from neonatal mice were pretreated with 3% hydrogen peroxide for 10 minutes at room temperature and rinsed 3 times 3 minutes in PBS, followed by antigen retrieval with Citra Plus buffer (BioGenex) for 30 minutes at 97°C. Lung sections were incubated with a nonspecific protein-blocking solution containing animal serum (Vector Laboratories) overnight at 4°C with the primary antibody. Lung sections were rinsed 3 times 2 minutes in PBS and incubated with secondary goat anti-rabbit IgG antibody (1:200 in PBS) for 30 minutes at room temperature. Immunoreactivity was visualized using the Vectastain ABC HRP Kit (Vector Laboratories) and the 3,3′-diaminobenzidine (DAB) substrate kit (Vector Laboratories). After visualization of the reaction, the sections were counterstained with H&E (Sigma-Aldrich) for 5 seconds, dehydrated, cleared, and mounted as described above.

### μCT scanning.

The trachea was tied in order to preserve a residual volume of air in the lungs. Then, the thoracic cavity was longitudinally opened and the thymus was removed to easily access the heart. The right ventricle was cannulated in the direction of the pulmonary artery using a 22-gauge cannula (Venisystems) and the left atrium was cut. Lungs were perfused with preflush solution (Ringer solution, B Braun) supplemented with 5% Dextran 70 (Roth), 5 IU/mL heparin (Ratiopharm), and 0.02% lidocaine (Jenapharm) for 15 minutes to remove the circulating blood. The preflush solution was switched to fixative (25% polyethylene glycol 400, 10% ethanol, and 3.7% formaldehyde [all from Roth]) for 30 minutes at 20 cm fixative pressure. Then, lungs were stained with osmium as previously described (49). Osmium-stained lungs were scanned using the SkyScan 1176 (Bruker microCT). Images were obtained in the absence of any contrast agent with the following settings: 50 kV, 500 μA source current, 0.5 mm aluminum filter, 9 μm resolution, and a rotation range of 180°. Reconstruction and analyses of the scans were performed with NRecon program (version 1.7.0.4, Burker microCT). The mouse airway tree was determined as previously described (50) and each airway generation was evaluated for mucus obstruction, using a numeric system from 0 to 2 (0 = non, 1 = mild and 2 = severe airway mucus obstruction). Researchers were blind to the genotype. Results are shown as the mean of the whole lung.

### Lung liquid content.

Lungs were removed from the chest and weighed (wet weight), incubated for 48 hours at 72°C, and weighed again (dry weight). Results are shown as the ratio between wet and dry weight.

### Real-time RT-PCR.

Dissected lungs were immediately stored in RNA later (Applied Biosystems). RNA was isolated using TRIzol (Invitrogen). cDNA was obtained by reverse transcription of 1 μg of total RNA with Superscript III RT (Invitrogen). Real-time PCR was performed on an Applied Biosystems 7500 Real-Time PCR System using TaqMan universal PCR master mix and TaqMan gene expression assays for *Cftr* (Mm00445197_m1), *Tmem16a* (*Ano1*; Mm00724407_m1), *Slc26a9* (Mm00628490_m1), *Muc5b* (Mm00466391_m1), *Muc5ac* (Mm01276718_m1), and *Actb* (Mm02619580_g1) according to the manufacturer’s instructions (Applied Biosystems). Relative fold changes in target gene expression were calculated from the efficiency of the PCR reaction and the crossing point deviation between samples from the different genotypes in relation to WT controls and normalization to the expression of the reference gene *Actb* (51).

### Culture of tracheal explants.

Tracheal explant cultures were performed as previously described (22). Briefly, tracheas were dissected from newborn mice (up to 12 hours after birth), placed in petri dishes (Thermo Fisher Scientific) containing 1 mL of tracheal explant (TE) medium (F12 Nutrient Mixture [Ham’s] + L-glutamine [Gibco]), supplemented with 10% FBS (Gibco), 5 mg/mL penicillin/streptomycin (Gibco), and 5 mM HEPES buffer (Millipore), and incubated at 37°C in 5% CO_2_ and 95% O_2_. The genotype was determined within 48 hours. A mixture of 400 μL Matrigel Matrix high concentration (HC) (Corning) and 200 μL of cold TE medium was put into a bottom glass petri dish (Cellvis). WT or *Slc26a9^–/–^* tissue was added and gelation was performed according to manufacturer’s instructions. After solidification of the Matrigel Matrix, the tissues were covered with TE medium and incubated for a total of 6–8 days. Medium was changed every second day. At days 6–8 in culture, tracheal tissues formed a cyst-like structure filled with liquid secreted by the airway epithelial cells. At least 15 minutes before the experiment, the TE culture medium was replaced with Ringer solution (145 mM NaCl, 0.4 mM KH_2_PO_4_, 1.6 mM K_2_HPO_4_•3H_2_O, 5 mM glucose, 1 mM MgCl_2_•6H_2_O and 1.3 mM Ca-gluconate•1H_2_O). The transepithelial potential difference was continuously recorded using a voltmeter setup when the cyst has been impaled with a glass microelectrode (mean tip resistance 14.9 MΩ) filled with Ringer solution. The microelectrode was connected to a standard voltmeter and referenced to the bath solution. Impalements were accepted if they met the following criteria: (a) the electrode tip was seen to penetrate the explant wall, (b) baseline readings before and after impalement differed by less than 1 mV, (c) the recording was stable for at least 5 minutes, and (d) there was no visible hole in the cyst when the microelectrode was withdrawn (52). Data were analyzed using Lab chart (Lab Chart 7, ADInstruments Pty Ltd).

### Flow cytometry.

Multiparameter flow cytometry was performed with an LSRFortessa flow cytometer (BD Biosciences), as previously described (27). In brief, lungs were perfused with 2.5 mL PBS, removed, transferred to RPMI-1640 (GE Healthcare Life Sciences), placed in a petri dish, and minced with scissors in pieces no larger than 1 or 2 mm. After that, lung tissue was transferred into 10 mL of PBS containing 300 U/mL collagenase type II (Worthington Biochemical) and 0.15 mg/mL DNase I (Sigma-Aldrich) and incubated on a shaker with an orbital speed of 125 rpm for 30 minutes at 37°C. To obtain single-cell suspensions, the solution was passed through a 100 μm filter and 40 μm filter. Red blood cells were lysed with red blood cell lysis buffer (BD Biosciences), and single cells were resuspended in RPMI-1640 medium.

The remaining cells were incubated with Fc Block (BD Biosciences) on ice, before staining with specific antibodies. For detection of neutrophils (CD45^+^Ly6G^+^CD11b^+^), monocytes (CD45^+^CD11c^+^MHCII^+^), and eosinophils (CD45^+^SinglecF^+^CD64^–^), cells were incubated with CD45-V500, Siglec-F-R phycoerythrin (PE) (clone 30-F11, BD Biosciences), MHC class II–V450 (clone M5/114.15-2, BioLegend), CD11b-Qd605, Ly6G–(PerCP)-Cy5.5 (clone M1/70, BioLegend), CD3–Alexa Fluor 700 (clone 17A2, BD Biosciences), CD11c-APC, CD4–fluorescein isothiocyanate (CD4-FITC) (clone HL-3, BD Biosciences), and CD64-BV711 (clone X54-5/7.1, BioLegend) antibodies for 30 minutes at 4°C. Total cell numbers and viability were assessed by using the trypan blue exclusion test. Cells were fixed with intracellular fixation buffer to allow short-term storage. Flow cytometry was performed with exclusion of nonviable cells and doublets, and data were analyzed with FlowJo software (v10, TreeStar).

### ELISA and CBA.

Concentrations of KC (MKC00B), MIP-2 (MM200), IL-1α (MLA00), and IL-1β (MLB00C) were measured in whole lung homogenates by ELISA (R&D Systems) according to the manufacturer’s instructions. Concentrations of TNF-α (BD Biosciences, 562336) and IL-13 (BD Biosciences, 562273) were measured with the CBA Enhanced Sensitivity Flex Set (BD Biosciences) according to the manufacturer’s instructions.

### Microbiology.

Dissected lungs from newborn mice were homogenized in a FastPrep-24 5G homogenizer (MP Biomedicals), using 200 μL PBS and autoclaved 1.4 mm ceramic spheres (Lysing Matrix D bulk, MP Biomedicals). Each tissue was plated on Columbia blood agar plates (Becton Dickinson) and pre-reduced Schaedler agar (BioMérieux) and incubated at 37°C. Colony-forming units (CFUs) were counted after 48 hours.

### Statistics.

All data were analyzed with Prism 9.5.1 for Windows (GraphPad Software) and are reported as mean ± SEM. We performed statistical analyses using a 2-tailed Student’s *t* test, Mann-Whitney test, χ^2^ test, and Kaplan-Meier survival analysis as appropriate. A *P* value of less than 0.05 was accepted to indicate statistical significance.

### Data availability.

All data are available in the main text or the supplemental material. Values for all data points in graphs are reported in the Supporting Data Values file.

## Author contributions

The order of co–first authors, PMB and JJS, was determined based on contributions to the experiments and writing the original manuscript draft. PMB, JJS, AB, and MAM conceptualized the study. PMB, JJS, JD, SS, PA, WLW, MOW, HJG, AB, and MAM developed the methodology. PMB, JJS, JD, SS, PA, WLW, MOW, HJG, AB, and MAM conducted experiments. PMB, JJS, AB, and MAM generated figures. MAM acquired funding. JJS, JD, AB, and MAM supervised the study. PMB, JJS, AB, and MAM wrote the original draft of the manuscript, which was reviewed and edited by PMB, JJS, JD, SS, PA, WLW, MOW, HJG, AB, and MAM.

## Funding support

German Research Foundation (project no. 450557679; SFB 1449, project no. 431232613; and EXC 3118/1, project no. 533770413).

- German Federal Ministry of Research, Technology and Space (grants 82DZL00401, 82DZL004A1, 82DZL009C1 and 01GL2401A).

## Supplementary Material

Supplemental data

Supplemental video 1

Supplemental video 2

Supporting data values

## Version 1 10/17/2025

In-Press Preview

## Version 2 12/08/2025

Electronic publication
