# Supplementary material for: Lack of SLC26A9-mediated chloride secretion causes mucus plugging and severe respiratory distress in neonatal mice
Source: JCI Insight. 2025 Oct 16;10(23):e196355. doi: 10.1172/jci.insight.196355 (PMC12890477; doi:10.1172/jci.insight.196355)
Supplement: Supplemental data [file jciinsight-10-196355-s222.pdf]

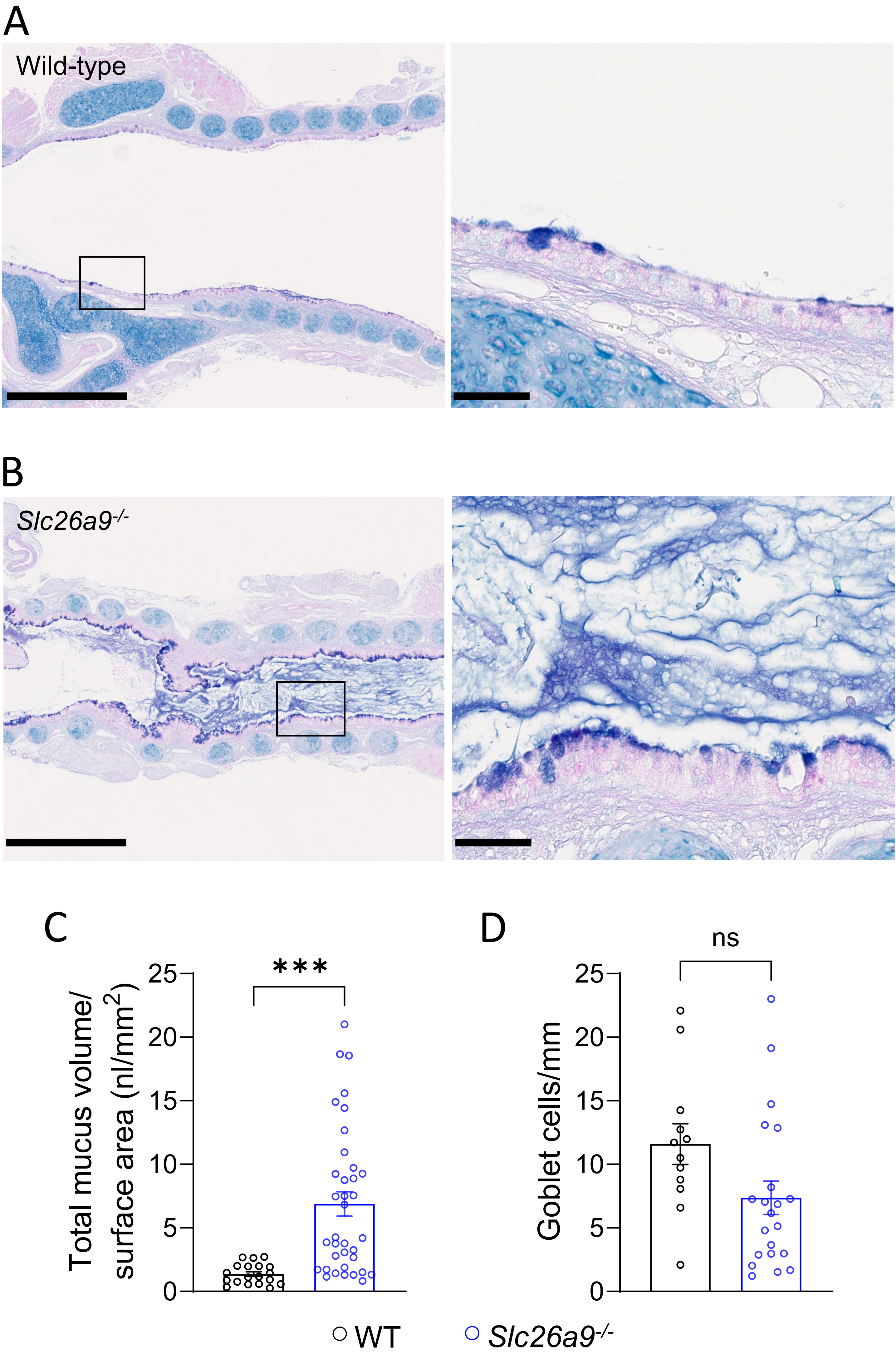

Supplemental Figure 1. Quantification of goblet cells and intraluminal mucus content in the trachea of newborn wild-type (WT) and *Slc26a9*$^{-/-}$ mice. (A, B) Representative images of AB-PAS stained trachea sections from newborn WT and *Slc26a9*$^{-/-}$ mice. Scale bar: 500 µm, inlets 50 µm. (C) Total mucus content and (D) goblet cell counts in the trachea of newborn WT and *Slc26a9*$^{-/-}$ mice. N = 12 – 36 mice per group. *** $p < 0.001$

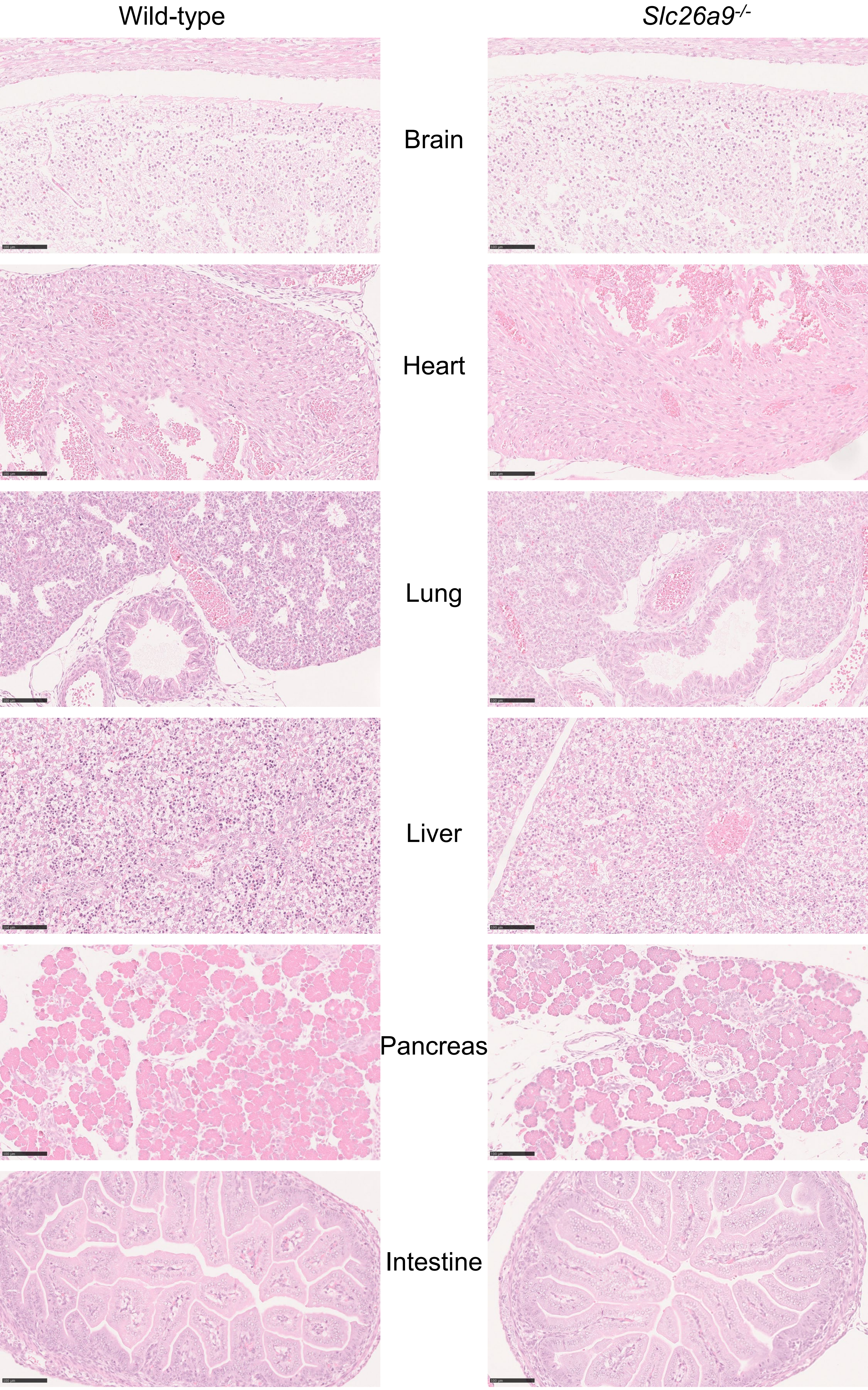

Supplemental Figure 2. Histopathological analysis of vital tissues revealed no changes in *Slc26a9*$^{-/-}$ mice at E17. Representative images of vital tissues, including brain, heart, lung, liver, pancreas and intestine in wild-type (n=3) and *Slc26a9*$^{-/-}$ (n=5) mice. Scale bar: 100 μm.

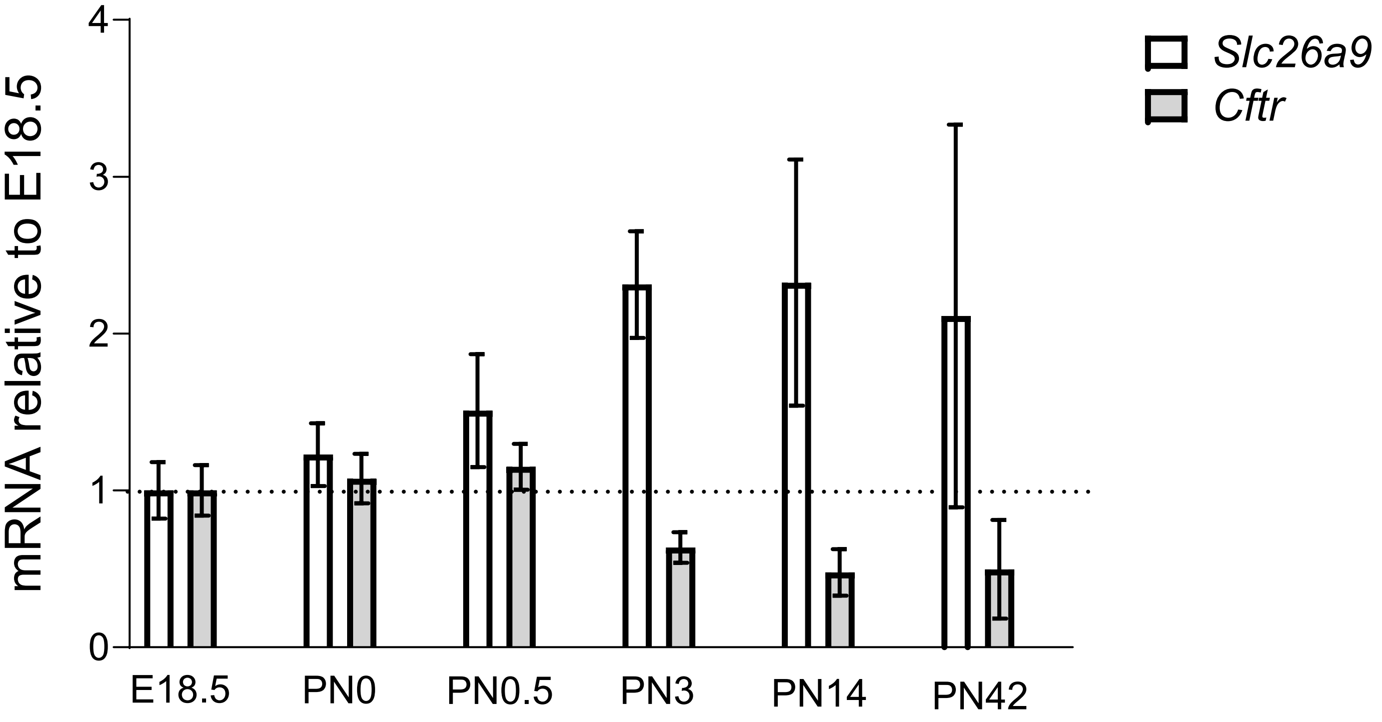

Supplemental Figure 3. Transcript expression of *Slc26a9* and *Cftr* in wild-type mouse lung homogenates during development. N = 4 – 16 mice per group.
